# Investigating the Quality of Mobile Apps for Drug-Drug Interaction Management Using the Mobile App Rating Scale and K-Means Clustering: Systematic Search of App Stores

**DOI:** 10.2196/65927

**Published:** 2025-10-13

**Authors:** Ayush Bhattacharya, Jose Fernando Florez-Arango

**Affiliations:** 1 Department of Population Health Sciences, Weill Cornell Medicine, 425 E 61st, Room 323, New York, NY, 10027, United States, 1 979 481 7392

**Keywords:** drug interactions, Mobile App Rating Scale (MARS), mobile health (mHealth), K-Means clustering, correlation analysis, app quality assessment, digital health, mobile app, United States

## Abstract

**Background:**

Drug-drug interactions (DDIs) pose a significant risk to patient safety and increase health care costs. Mobile apps offer potential solutions for managing DDIs, yet their quality and effectiveness from the user’s perspective remain unclear.

**Objective:**

The aim is to evaluate the quality of publicly available mobile apps for DDI management in the US using the Mobile App Rating Scale (MARS) and to identify patterns that reflect user satisfaction and preferences.

**Methods:**

A structured review was conducted to identify mobile apps for DDI management, resulting in 19 eligible apps. Two health care–affiliated evaluators independently assessed each app using the mobile app rating scale (MARS). Dimensionality scores were calculated, and correlation analysis was conducted to examine relationships among dimensions. K-means clustering was applied to group apps based on their MARS scores. Scatter plots visualized app distributions across clusters. To validate the clustering model and assess alignment with user satisfaction, mean weighted user ratings were compared with mean MARS scores per cluster. Correlation analysis was also performed between individual MARS dimensions and user ratings within each cluster.

**Results:**

The mean MARS score was 3.54 out of 5, with the Information dimension scoring the highest (mean 3.68, SD 0.51) and Engagement the lowest (mean 3.42, SD 0.80). The Kruskal-Wallis test revealed no significant differences in median scores across the four dimensions (*χ*²_3_=2.109, *P*=.55). All MARS dimensions were positively correlated (*r*=0.65 to 0.92), indicating interrelated quality characteristics. K-means clustering identified three app groups with varying quality profiles: Cluster 1 (n=7, mean MARS=2.86), Cluster 2 (n=7, mean=3.57), and Cluster 3 (n=5, mean=4.44). Cluster 1 apps showed strongest correlations between user satisfaction and functionality (*r*=0.74) and engagement (*r*=0.53). Cluster 2 users prioritized information (*r*=0.41) and aesthetics (*r*=0.58), and Cluster 3 exhibited balanced influence from information (*r*=0.62), aesthetics (*r*=0.58), and functionality (*r*=0.39). Scatter plots indicated that engagement, functionality, and aesthetics were key drivers of user perception, while information, though consistently strong, played a lesser role in differentiating the apps. The weighted user ratings aligned with MARS scores, supporting the validity of the clustering model.

**Conclusions:**

This study assesses the quality of mobile apps for DDI management by integrating MARS with K-means Clustering. This approach enabled a structured classification of apps based on the MARS scores, identifying distinct clusters that reflect overall app quality profiles across key usability dimensions. The study revealed that the influence of MARS dimensions on app ratings varies by cluster, highlighting that the significance of these dimensions shifts according to the specific needs and preferences of different user groups.

## Introduction

Drug-drug interactions (DDIs) pose significant risks to patient safety and contribute substantially to adverse drug reactions, which can result in serious health complications and increased health care costs. DDIs occur when the pharmacological or clinical response to a drug is altered by the presence of another drug, potentially leading to harmful effects [1]. In the United States, DDIs account for approximately 26% of adverse drug reaction–related hospital admissions [2], highlighting the critical need for effective DDI management strategies.

The advent of mobile health (mHealth) applications has introduced new possibilities for managing DDIs by providing decision support tools directly to consumers and health care professionals. These apps can facilitate the detection, avoidance, and reporting of potential DDIs, thereby enhancing medication safety and improving patient outcomes. The increasing availability and use of smartphones have made these tools accessible to a broad audience, including older adults who are often at higher risk for medication errors due to polypharmacy [3].

Despite the growing availability of mHealth apps for DDI management, their quality and usability vary significantly. Previous studies have assessed app quality using the mobile app rating scale (MARS) and found that while most apps scored well on information and functionality, they often lacked engaging features and consistent usability. For instance, Kim et al [4] and Shen et al [5] both reported that the information and functionality dimensions were rated the highest, while engagement consistently received the lowest scores. However, these studies relied primarily on descriptive MARS scores and did not explore how different quality dimensions influence user satisfaction or reveal patterns in app quality.

Building on these insights, our study aims to evaluate the quality of publicly available mobile apps for DDI management in the United States using the MARS and to identify patterns in user preferences [6-8]. Unlike previous studies that relied on descriptive MARS summaries, we employ k-means clustering, an unsupervised machine learning technique that has not been previously applied for MARS analysis before. This innovative approach enabled the identification of clusters of apps with similar quality profiles and helped uncover patterns in how MARS dimensions relate to user preferences. By linking expert-based MARS scores with real-world app store ratings, this study offers a more user-centered perspective and provides practical insights to guide future app development and evaluation.

## Methods

### App Selection Process and Distribution

The app selection process conducted in this study followed the Preferred Reporting Items for Systematic Reviews and Meta-Analyses (PRISMA) protocol [9] as closely as possible. Although this was not a formal systematic review, our methodology was aligned with PRISMA principles, and the checklist was followed where applicable to ensure transparent and robust reporting of methods and results. Some deviations from PRISMA were necessary due to the distinct characteristics of mHealth app store databases, which were dissimilar from conventional scholarly reference databases. App store search algorithms are not transparent, and results may vary over time, limiting reproducibility.

The app identification process followed a structured approach to search for relevant apps using specific keywords (Table 1) related to drug interactions in the Apple App Store and Google Play Store. The search was conducted in March 2024 on a designated set of devices to ensure uniformity of results. All searches were performed on devices physically located in the United States using US-registered Apple ID and Google Play accounts to ensure inclusion of apps available in the US market only. The identified apps were screened for eligibility in 2 stages. First, the app descriptions were reviewed to determine if they claimed to check for DDIs and were published in English. Second, apps were excluded if they were not intended for general consumers, did not allow for multiple or combination interaction checks, were designed for pets or animals, or were specific to a particular disease or drug class. The selected app names were compiled, and the apps were downloaded and installed to verify their eligibility. A total of 19 apps met the inclusion criteria and were selected for subsequent analysis. User ratings and the number of reviews for these apps were recorded from the respective app stores. All data processing and statistical analyses were conducted using the latest version of RStudio.

The quality of the mHealth apps was assessed using MARS, a 23-item expert-based rating scale designed to evaluate various aspects of app quality [10]. The MARS scale provides a 5-point rating system to assess quality dimensions, such as end-user engagement, features, aesthetics, information, and subjective quality. In this study, we excluded the subjective quality dimension of the MARS scale. This decision was made to maintain the objectivity and consistency of our evaluation, as the subjective quality dimension is inherently more prone to bias, even among evaluators with similar professional backgrounds. Two evaluators independently assessed each app. Both were graduates of Weill Cornell Medicine with health care-related backgrounds and substantial experience in digital health. One evaluator is a licensed physician with clinical experience and advanced training in medical informatics. Another evaluator holds a master’s degree in Biostatistics and has over five years of professional experience working with health data in research and industry settings. Their combined expertise ensured both clinical insight and technical rigor in evaluating app content, usability, and functionality. The interrater solid reliability (IRR) reflecting the degree of agreement [11] between the two evaluators was estimated using Cohen’s kappa (κ) statistic. The IRR was calculated per dimension and for all apps, using a two-way random model for agreement level.

**Table 1.** Search Terms with REsulTS.

Search Term	Raw Results	Results After Exclusion Criteria
“Drug-Drug Interaction”	70	12
“Drug Checker”	56	5
“Medication Tracker”	145	1
“Drug Interaction”	85	6
“Drugs Interaction”	69	12
“Drug Interactions”	83	10
“Drugs Interactions”	83	10
“Drug-Interaction”	81	11
“Pill Interaction”	1	0
“Pills Interaction”	0	0
“Pill Interactions”	0	0
“Pills Interactions”	0	0
“Pill-Interaction”	1	0
“Medication Interaction”	18	2
“Medications Interaction”	0	0
“Medication Interactions”	105	4
“Medications Interactions”	2	1
“Medication-Interaction”	18	2

To understand the relationship between the different MARS dimensions, we first assessed the distribution of the data. We used quantile-quantile (Q-Q) normal plots to evaluate this. The Q-Q plots helped us to visually determine how well the data for each dimension fit a standard normal distribution. The Q-Q plots helped us to determine deviations from normality; therefore, we performed the Kruskal-Wallis test to compare the dimension groups and draw further inferences. The Kruskal-Wallis test is a non-parametric method used to determine whether there are differences between two or more groups by examining differences in their median values. We then performed correlation analysis to understand the strength and direction of the linear relationships between each pair of dimensions. The correlation coefficient ranges from −1 to 1, where 1 indicates a perfect positive correlation, 0 indicates no correlation, and −1 indicates a perfect negative correlation.

**Figure 1.**
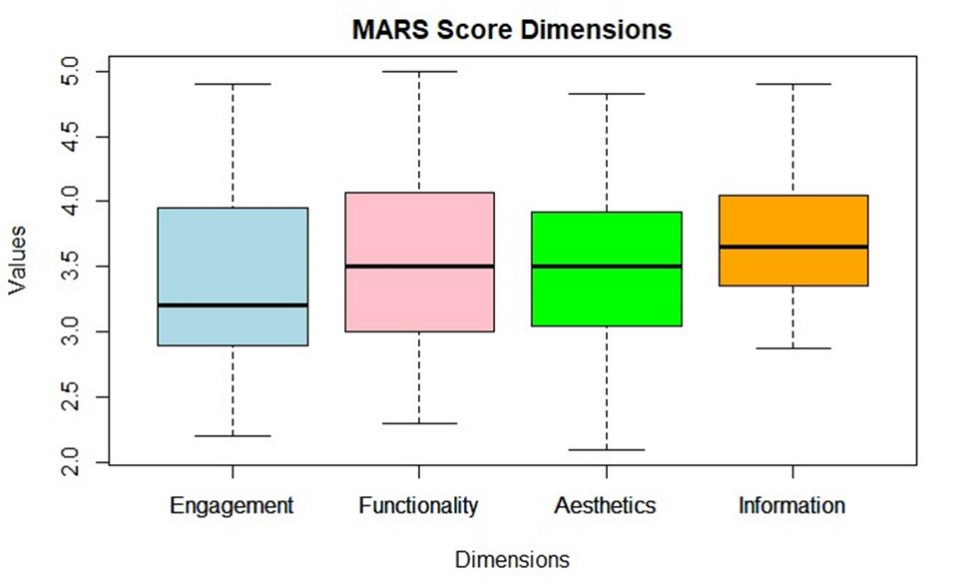
Mars Dimensions Score Box PloT. Mars: Mobile App Rating Scale.

We then categorized the apps into distinct groups based on their MARS dimension scores using k-means clustering [12]. This unsupervised machine learning technique groups similar data points into distinct clusters, helping us identify patterns and relationships between the dimensions and group apps with similar characteristics. The first step in k-means clustering is to determine the optimal number of clusters. We used the elbow plot approach to identify this number [13]. The elbow plot shows the number of clusters against the within-cluster sum of squared errors (SSE). The plot shows a decreasing SSE as the number of clusters increases. The “elbow” point on the plot indicates where the rate of decrease in SSE slows down and becomes less pronounced. This point represents the optimal number of clusters, suggesting that adding more clusters does not significantly improve the clustering solution. While commonly used, this approach involves some degree of visual interpretation and subjectivity, and the inherent number of clusters may influence the resulting groupings. After identifying the optimal number of clusters, we then perform the k-means clustering (Figure 1).

After k-means, to further explore and visualize the clusters, we used scatter plots. The scatter plots allowed us to visually examine the distribution of MARS scores across different dimensions and understand the clustering patterns more clearly. By plotting the dimensions against each other, we identified how the clusters are separated and discerned relationships and trends within and between the clusters, providing a more intuitive understanding of the data.

Following this, we integrated user ratings and the number of reviews from both the Google Play Store and Apple App Store to validate our k-means clustering results with user feedback. To provide a more accurate estimate of app ratings, we calculated a weighted average that considers both the ratings and the number of reviews on each platform (Table 2). We rounded the number of reviews to the nearest multiple of 5. To evaluate the effectiveness of our clustering model, we compared the mean of weighted average ratings for all apps within each cluster with the mean MARS score for that cluster. This approach allowed us to validate our clustering methodology, ensuring it produces meaningful results that align with actual user experiences and preferences.

**Table 2.** Ratings and Review COunts for APps on Google Play Store and Apple App Store

App Name	Google Play Store		Apple App Store		Weighted Average Rating
	Rating	Number of Ratings	Rating	Number of Ratings	
Apothera	0	0	4.3	15	4.30
Avicenna - Drug Interactions	0	0	0	0	0.00
Drug Compatibility Checker Tuh	1	25	0	0	1.00
Drug Interaction Checker +	4.7	145	4.1	60	4.52
Drugs.com Medication Guide	4.6	32,600	4.8	8600	4.64
Elsevier Clinical Pharmacology	3.3	400	2.6	5	3.29
Epocrates	4.1	26,500	4.5	6700	4.18
Everydose Medication Reminder	4.3	1250	4.8	3500	4.67
Goodrx	4.8	298,000	4.8	652,000	4.80
Lexidrug	3.3	3300	3.1	150	3.29
Medisafe Medication Manager	4.6	240,000	4.7	90,000	4.63
Medscape	4	635,00	3.6	1250	3.99
Micromedex Drug Interactions	4.1	500	3.8	100	4.05
Myrxprofile	5	85	3.6	25	4.68
Pill Identifier & Drug Search	3.9	1000	4.2	100	3.93
Pillbox	3.3	630	3.8	85	3.36
Pocket Pharmacist	4.5	11,770	4.7	3535	4.55
The Washington Manual	4.7	155	4.7	180	4.70
Uptodate	4	10,800	3.9	450	4.00

We then used quantile-quantile (Q-Q) plots to evaluate the distribution patterns of the Weighted Average Ratings and the MARS Mean Scores. The Q-Q plots revealed deviations from normality, and therefore, we used the paired Wilcoxon signed-rank test to compare these two metrics within each cluster. The Wilcoxon signed-rank test is a nonparametric test used to compare the median of two related samples when the data does not follow a normal distribution [14,15]. We chose the paired version of this test because the measures being compared—weighted average ratings and MARS scores—are evaluations of the same group of apps [16].

Additionally, to further explore the relationship between the MARS dimensions and the weighted average ratings within each cluster, we conducted a correlation analysis. This analysis aimed to identify the specific MARS dimensions that align closely with user ratings, as well as those that do not, thus providing a deeper understanding of how MARS dimensions relate to user perceptions.

### Ethical Considerations

This study did not involve human participants, patient data, or identifiable information. The analysis was limited to publicly available mobile applications and app store metadata (descriptions, features, ratings). Institutional review board (IRB) approval was not required, and informed consent was not applicable.

## Results

### App Identification and Selection

**Figure 2.**
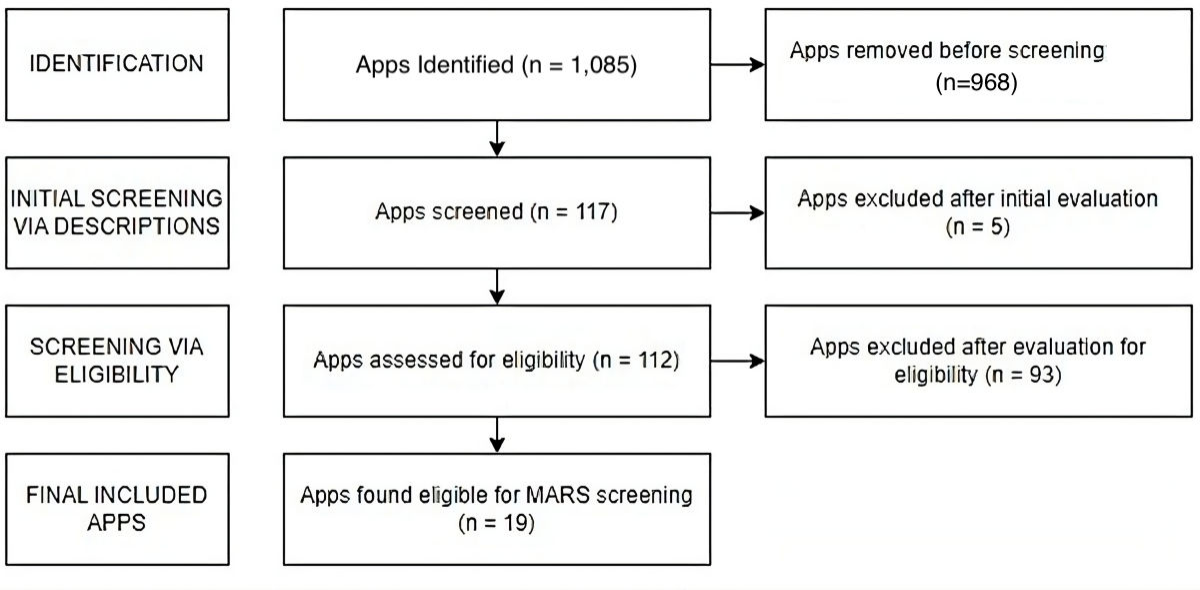
App Selection PRocesS. Mars: Mobile App Rating Scale.

The app store search identified a total of 1085 apps from the Google Play Store and Apple App Store. After removing duplicates, 117 unique apps remained for screening. Based on their descriptions and functionality, 19 apps met all inclusion and exclusion criteria and were selected for full evaluation (Figure 2); a completed PRISMA checklist is provided in Checklist 1.

### Overall Scores

The mean MARS score of the 19 apps was 3.54. Some notable apps with high ratings were Medisafe Medication Manager, GoodRx, and Drugs.com Medication Guide (Table 3). On the other hand, Elsevier Clinical Pharmacology and Drug Compatibility Checker TUH received relatively low ratings across all categories. The IRR between two raters as assessed by the kappa statistic was 0.46.

The mean scores of the four dimensions of MARS were examined to investigate the magnitude of the differences in quality in each dimension (Table 4). The information dimension resulted in the highest mean and median scores (3.68 and 3.65 (IQR 3.35-4.05), respectively), whereas the engagement dimension showed the lowest mean score (3.42) and lowest median score (3.21 (IQR 2.90 ). These findings align with observations in similar studies [4,5]. The aesthetics dimension also had the most variability (SD 0.81).

**Table 3.** Apps and Their Mars^a^ ScoreS.

App Name	Engagement	Functionality	Aesthetics	Information	Mean Mars Score
Apothera	3.10	4.00	3.83	3.40	3.58
Avicenna - Drug Interactions	3.00	3.63	3.00	3.00	3.16
Drug Compatibility Checker Tuh	2.45	2.90	2.67	2.87	2.72
Drug Interaction Checker +	3.80	3.30	3.75	3.60	3.61
Drugs.com Medication Guide	4.10	4.75	4.20	4.30	4.34
Elsevier Clinical Pharmacology	2.20	2.30	2.15	2.95	2.40
Epocrates	4.50	4.38	4.00	3.80	4.17
Everydose Medication Reminder	3.80	3.75	3.40	4.10	3.76
Goodrx	4.85	5.00	4.75	4.14	4.69
Lexidrug	2.90	3.10	3.30	3.12	3.11
Medisafe Medication Manager	4.90	5.00	4.83	4.75	4.87
Medscape	3.30	3.45	3.50	3.70	3.49
Micromedex Drug Interactions	2.60	2.72	2.10	3.65	2.77
Myrxprofile	3.10	2.90	3.12	3.30	3.11
Pill Identifier & Drug Search	2.60	2.70	2.10	3.60	2.75
Pillbox	3.21	3.45	3.53	3.87	3.52
Pocket Pharmacist	4.20	4.13	4.17	4.00	4.13
The Washington Manual	2.90	3.80	3.10	4.24	3.51
Uptodate	3.40	3.50	3.70	3.60	3.55

a Mars: Mobile App Rating Scale.

**Table 4.** Mars^a^ Dimension SCoreS.

Dimensions	Mean Ratings	Median Ratings (Iqr)	Standard Deviations
Engagement	3.42	3.21 (2.90 -3.95)	0.80
Functionality	3.62	3.50 (3.0 - 4.06)	0.78
Aesthetics	3.43	3.50 (3.05 - 3.91)	0.81
Information	3.68	3.65 (3.35 - 4.05)	0.51

a Mars: Mobile App Rating Scale.

**Figure 3.**
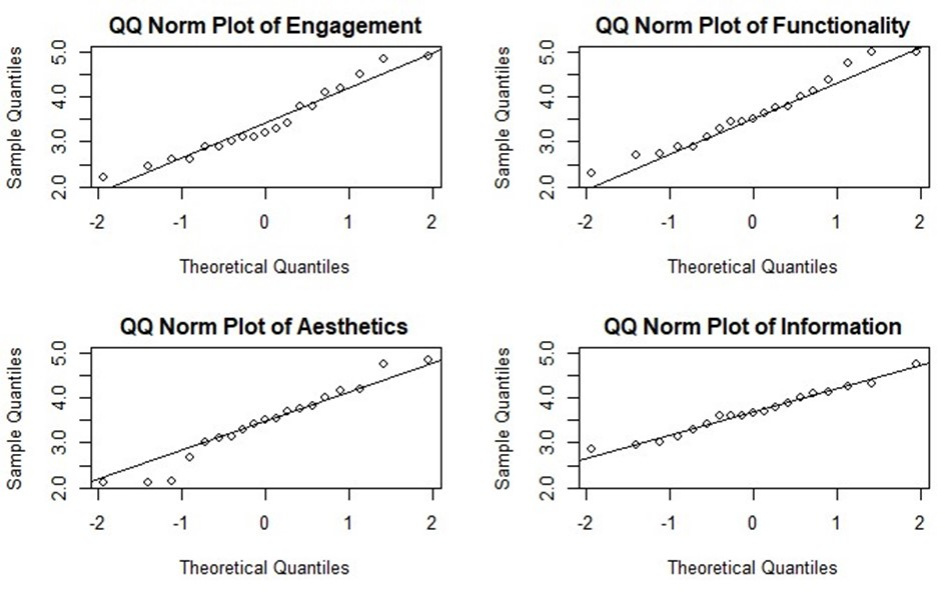
Quantile-Quantile Normal Plots of Mars Dimensions. Mars: Mobile App Rating Scale.

The Q-Q plots (Figure 3) reveal that engagement, functionality, and aesthetics dimensions deviate from a standard normal distribution, particularly at the tails, indicating the presence of more extreme values than expected under normality. In contrast, the information dimension adheres more closely to a normal distribution, with some minor deviations.

Since the dimension distributions are not uniformly normal, we perform the Kruskal-Wallis test. The null hypothesis is that the medians of all groups are equal, indicating no difference in the central tendency of these dimensions. The alternative hypothesis is that at least one dimension’s median is different from the others. The significance level is taken as .05. The test resulted in a *χ*^2_3_^ value of 2.109 and a *P* value of .55.

Since the *P* value is greater than .05, we have sufficient evidence to accept the null hypothesis that there is no significant difference between the median values of the dimension groups for engagement, functionality, aesthetics, and information. Therefore, there is no need to treat any dimension differently in terms of interventions, improvements, or prioritization based on median scores alone.

### Correlation Between Dimensions

**Figure 4.**
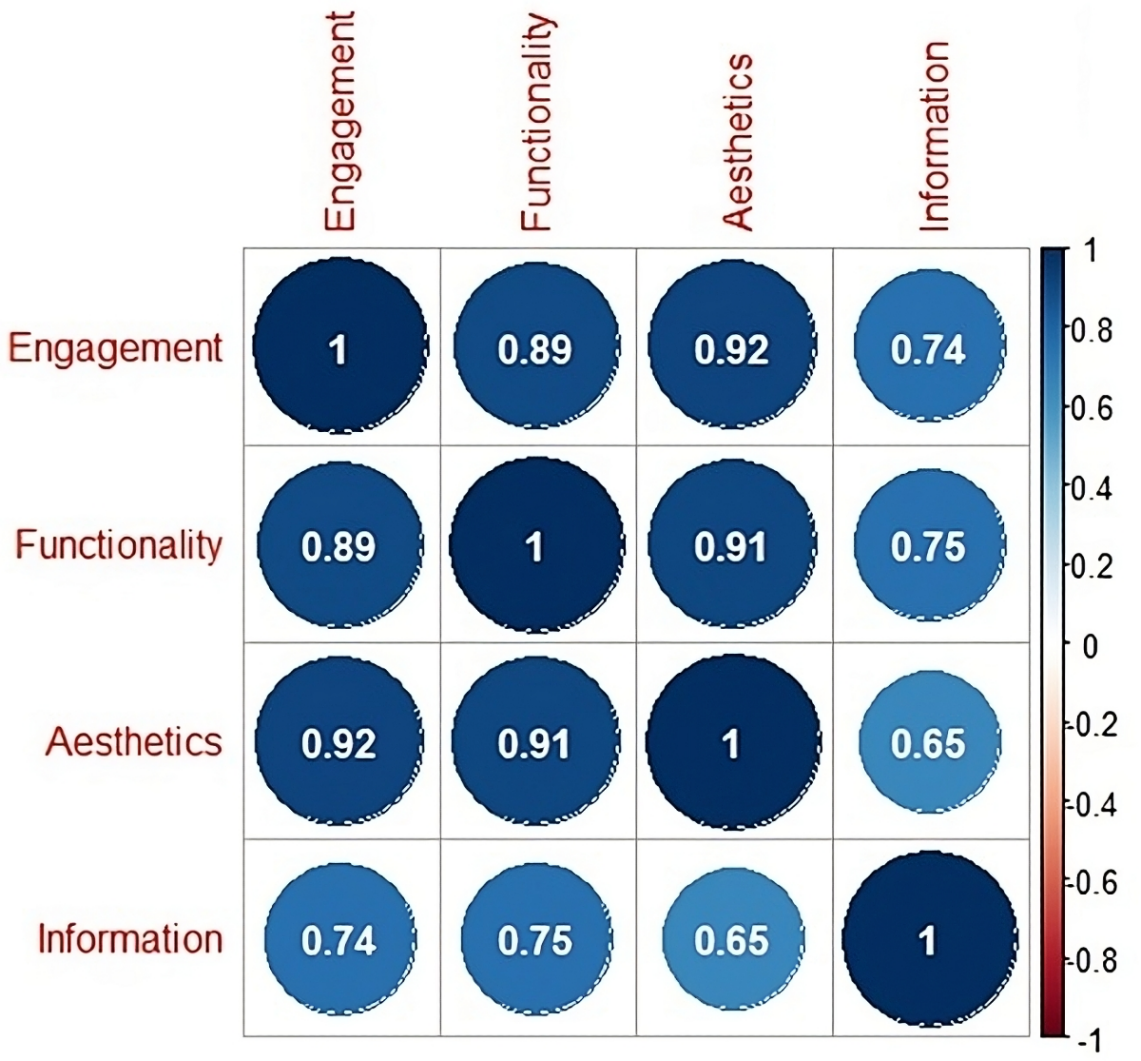
Correlation Plot of Mars DImensionS. Mars: Mobile App Rating Scale.

From the correlation plot (Figure 4), we see that all the correlation coefficients are positive, indicating a positive association between the dimensions. The strongest correlation is between aesthetics and engagement, with a coefficient of 0.92, followed closely by functionality and aesthetics with a coefficient of 0.91. The weakest correlation is between aesthetics and information, with a coefficient of 0.65.

### K-Means Clustering of Dimensions

Given the above findings, we apply K-means clustering to categorize the apps into distinct groups based on their MARS dimension scores.

**Figure 5.**
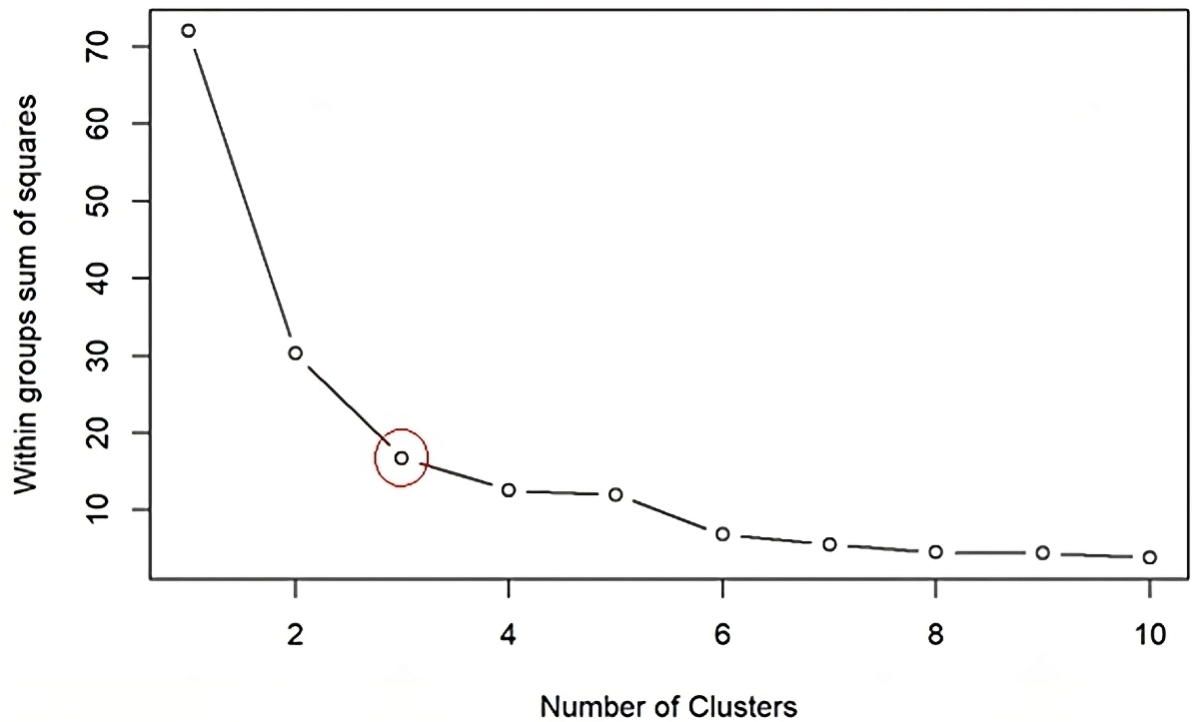
Elbow Plot to Identify the Optimal Number of ClusterS.

From the elbow plot (Figure 5), we see that 3 clusters are optimal for our study. We then run the k-means clustering, and each app is assigned to a cluster based on characteristics derived from the dimensions.

We see that cluster 3 apps consistently have the highest mean scores across all dimensions: engagement (4.51, SD 0.36), functionality (4.65, SD 0.39), aesthetics (4.39, SD 0.37), and information (4.20, SD 0.36; Table 5). This indicates that apps in cluster 3 generally perform exceptionally well across all dimensions. In contrast, cluster 2 apps show moderate mean scores for engagement (3.36, SD 0.34), functionality (3.61, SD 0.25), aesthetics (3.54, SD 0.25), and information (3.79, SD 0.30). Cluster 1 apps exhibit the lowest mean scores for engagement (2.69, SD 0.32), functionality (2.89, SD 0.41), aesthetics (2.63, SD 0.52), and information (3.21, SD 0.31), indicating lower overall performance.

**Table 5.** Apps in Cluster and the Mean and STandard DEviation (Sd) Scores by ClusteR.

Cluster	App Name	Engagement		Functionality		Aesthetics		Information	
		Mean	Sd	Mean	Sd	Mean	Sd	Mean	Sd
1	Avicenna - Drug Interactions, Myrxprofile, Pill Identifier & Drug Search, Micromedex Drug Interactions, Lexidrug, Elsevier Clinical Pharmacology, Drug Compatibility Checker Tuh	2.69	0.32	2.89	0.41	2.63	0.52	3.21	0.31
2	The Washington Manual, Everydose Medication Reminder, Drug Interaction Checker +, Apothera, Uptodate, Medscape, Pillbox	3.36	0.34	3.61	0.25	3.54	0.25	3.79	0.30
3	Goodrx, Drugs.com Medication Guide, Medisafe Medication Manager, Pocket Pharmacist, Epocrates	4.51	0.36	4.65	0.39	4.39	0.37	4.20	0.36

**Figure 6.**
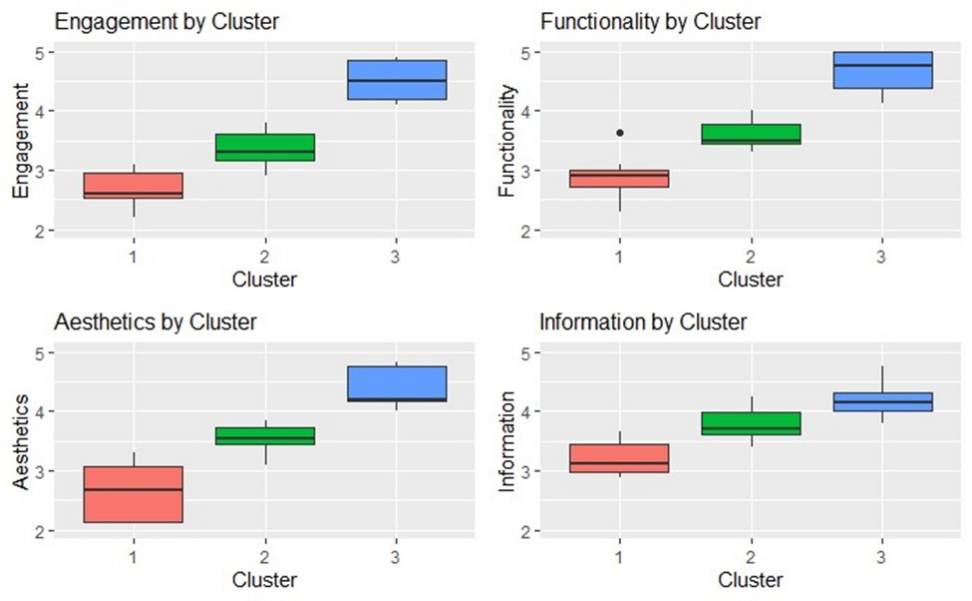
Box Plot of Mars Dimension Scores by ClusterS. Mars: Mobile App Rating Scale.

From the box plots (Figure 6), we see that cluster 3 apps show consistently high median scores and minimal variability, indicating well-rounded apps. Cluster 2 has moderate medians with some variability, particularly in functionality and aesthetics, indicating a solid performance with certain areas that may need improvement. Cluster 1 exhibits the lowest medians and greatest variability, especially in aesthetics and engagement. This highlights a diverse performance within cluster 1, where some apps may have specific strengths, but overall, they are of lower quality compared to those in clusters 2 and 3.

**Figure 7.**
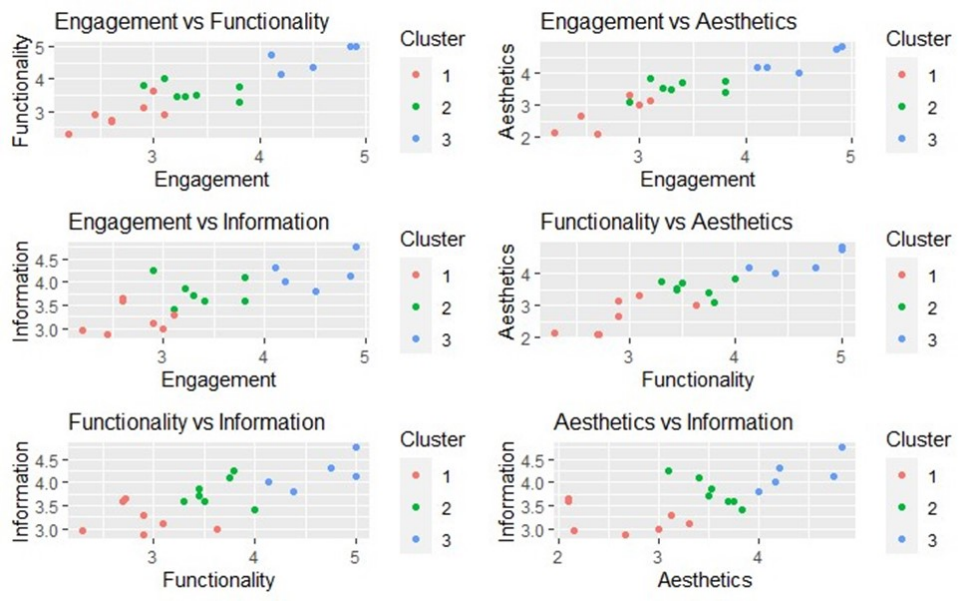
Scatter Plots of DImensionS.

To further explore and visualize these clusters, we use scatter plots to visually examine the distribution of app scores across different dimensions and understand the clustering patterns more clearly (Figure 7).

Cluster 3 (blue) consistently occupies the higher end of the scale in most dimensions, indicating that apps in this cluster are perceived to be of higher quality overall. Cluster 2 (green) apps show moderate ratings, occupying the middle range across dimensions. In contrast, cluster 1 (red) apps are positioned towards the lower end of the scale.

### Model Evaluation Based on User Ratings

To validate our K-means clustering model, we use the weighted app ratings. We first calculate the mean of weighted average ratings of all apps in the cluster and compare it with the mean MARS score of the cluster (Table 6).

Cluster 1 has the lowest mean weighted average rating of 3.30 and a relatively high standard deviation of 1.31, indicating lower user satisfaction with significant variability. This is mirrored by the mean MARS score, which is also the lowest at 2.86, suggesting that these apps are perceived as lower quality across the MARS dimensions, with a somewhat consistent perception reflected by the standard deviation of 0.28. In cluster 2, we see moderate user satisfaction, with a mean weighted average rating of 4.22 and a lower standard deviation of 0.64, indicating more consistent user experiences. This is closely aligned with the mean MARS score of 3.57, which, along with a very low standard deviation of 0.09, reflects a consistent perception of moderate app quality. Cluster 3 stands out with the highest mean weighted average rating of 4.84 and the lowest variability (SD=0.36), indicating that these apps are consistently well received by users. The high mean MARS score of 4.44, despite a slightly higher standard deviation of 0.33, reinforces the perception that these apps are of high quality across all MARS dimensions. To further substantiate these observations, we then proceeded to determine the statistical significance of the differences between the clusters.

**Figure 8.**
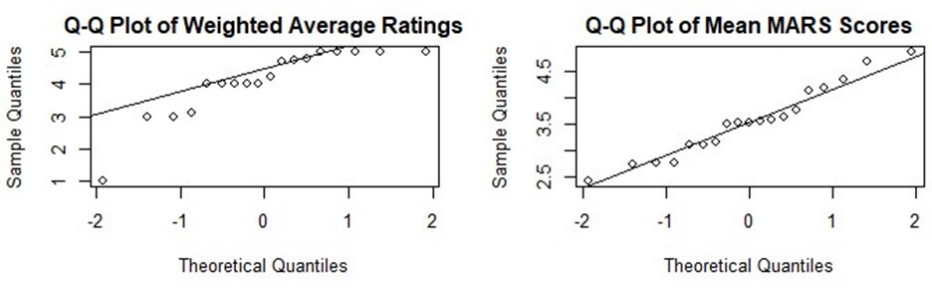
Q-Q Plots of Weighted Average Ratings and Mean Mars Scores. Mars: Mobile App Rating Scale.

The Q-Q plots for the weighted average ratings and mean MARS scores (Figure 8) show significant deviations from the reference line, indicating that the distribution of these ratings deviates from normality. Hence, we perform the paired Wilcoxon signed-rank test to compare the weighted average ratings and MARS scores. The null hypothesis is that there is no difference in the median values between the weighted average ratings and the MARS scores for the same apps. The alternative hypothesis is that there is a difference in the median values, and the significance level is taken as 0.05.

**Table 6.** Comparison of Weighted Average Ratings and Mars^a^ Score by ClusteR.

Cluster	Mean of Weighted Average Ratings	Median of Weighted Average Ratings	Sd of Weighted Average Ratings	Mean Mars Score	Median Mars Score (Iqr)	Sd of Mars Score	Test Statistic (V) of The Wilcoxon Signed-Rank Test	*P* VAlue
1	3.30	3.50	1.31	2.86	2.77 (2.74-3.11)	0.28	14	.56
2	4.22	4.00	0.64	3.57	3.55 (3.51-3.60)	0.09	27	.03*^b^
3	4.84	5.00	0.36	4.44	4.34 (4.17-4.69)	0.33	15	.06

a Mars: Mobile App Rating Scale.

b *P*<.05

The Wilcoxon signed-rank tests (Table 6) revealed distinct variations in the relationship between weighted average ratings and MARS scores across the clusters. In cluster 1, the *P* value of .56 suggests that there is no statistically significant difference between the median weighted average ratings and the median MARS scores, indicating that these two metrics are fairly aligned within this cluster. For cluster 2, however, the *P* value of .03 is below the significance threshold of .05, indicating a statistically significant difference between the two medians. This suggests that the weighted average ratings and MARS scores in cluster 2 may reflect different aspects of app quality or user perceptions. This could imply that the factors contributing to higher or lower user ratings on app stores may differ from those emphasized by the MARS criteria. In cluster 3, the *P* value of .06 is slightly above the .05 threshold, suggesting a marginal difference between the medians that needs more exploring.

**Figure 9.**
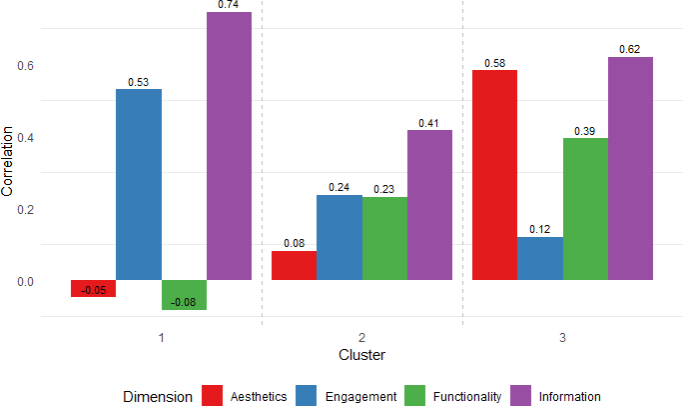
Correlations Between The Weighted Average Rating and Mars Dimension Scores by CLusteR. Mars: Mobile App Rating Scale.

To further understand the reasons behind these observations, we conducted a correlation analysis between the MARS dimensions and weighted average ratings within each cluster (Figure 9). This analysis helped identify which specific MARS components align closely with user ratings and which do not, providing deeper insights into the facets of app quality that resonate with users compared to those emphasized by the MARS criteria.

We observed that for cluster 1 apps, information and engagement are key drivers of user satisfaction, while aesthetics and functionality have less influence. In cluster 2, information quality stood out as the most significant factor, with functionality and engagement having a more moderate impact. Cluster 3 showed that aesthetics and information are the primary factors influencing user ratings, with engagement playing a minimal role. These insights suggest that user priorities differ across clusters, with some clusters valuing functionality and engagement more, while others placing greater importance on aesthetics and information.

## Discussion

### Principal Findings and Comparison With Previous Works

This study evaluated the quality of 19 mobile apps for DDI management using MARS and applied K-means clustering to identify patterns in app quality and user satisfaction. The results showed that while the information dimension received the highest scores, engagement was consistently rated the lowest. Strong correlations were observed across MARS dimensions, suggesting that app quality is multidimensional and interrelated. Cluster analysis revealed 3 distinct app quality profiles, with variations in how specific dimensions aligned with user ratings, highlighting differences in user preferences and design priorities across app types.

Among the MARS dimensions, the information dimension received the highest scores, indicating that these apps generally provide comprehensive and reliable content. This aligns with the core function of DDI apps, which are designed to deliver accurate drug interaction information quickly and efficiently. This finding is consistent with prior studies on drug-related mHealth apps, which also identified information as the most consistently strong dimension [4,5].

In contrast, the engagement dimension scored the lowest and exhibited considerable variability, suggesting that many DDI apps struggle to retain user interest. This is not unexpected, as these apps are typically used as reference tools for quick, task-oriented interactions rather than prolonged engagement. As such, lower engagement scores may reflect their intended purpose rather than a design flaw. This trend was also observed in prior studies by Kim et al [4] and Shen et al [5], where engagement received the lowest or most variable scores among MARS dimensions. It also aligns with broader mHealth research, which shows that reference apps often score lower in engagement due to their transactional design focus [17,18].

The functionality dimension scored relatively high, which indicates that these apps generally offer effective and user-friendly features. This is crucial for DDI apps, as users expect reliable and straightforward tools to check drug interactions efficiently. The high functionality scores were seen to be in alignment with previous studies [4,5] and suggest that most of these apps succeed in delivering on their core promise: providing users with easily accessible information.

Finally, the aesthetics dimension showed moderate scores with noticeable variability. This suggests that while some apps are visually appealing and offer a pleasant user experience, others may fall short in terms of design and visual coherence. Aesthetics can influence user perceptions, even for utilitarian apps like those for drug interactions. Although it might not be the primary concern for users, a well-designed interface can enhance usability and overall satisfaction.

The Kruskal-Wallis test showed no significant difference between the median values of the dimensions. This suggests that while there are notable differences in variability and individual scores, the overall central tendency of these dimensions does not differ significantly, indicating that no single dimension consistently outperforms or underperforms the others.

The correlation analysis showed strong positive relationships among MARS dimensions, indicating that apps performing well in one area tend to perform well across others. This interrelatedness suggests that improvements in any one domain may contribute to overall app quality and higher user satisfaction. For example, an app that is engaging and visually appealing (high engagement and aesthetics scores) is more likely to also have good functionality and provide useful information (high functionality and information scores), leading to a higher overall rating. Conversely, an app that performs poorly in one or more dimensions is more likely to have a lower overall rating. Therefore, app developers and designers should strive to create apps that perform well in all dimensions, as this is likely to lead to higher user satisfaction and better ratings.

The K-means clustering analysis revealed 3 distinct app groupings based on MARS dimension scores. Cluster 3 apps demonstrated high quality and consistency across all dimensions; cluster 2 showed moderate performance with variability; and cluster 1 included underperforming apps with the greatest variation. These groupings were supported by box plots showing clear differences in overall score distributions. Scatter plot analysis showed distinct clustering patterns for engagement, functionality, and aesthetics—dimensions that most strongly differentiated app quality. Cluster 3 apps consistently scored high across these, while cluster 1 scored lowest. However, plots involving information showed more overlap, particularly between clusters 2 and 3, indicating that information quality alone was less effective in distinguishing apps. This suggests that engagement, aesthetics, and functionality play a more significant role in shaping user perceptions.

We validated our clustering model by comparing mean MARS scores with weighted user ratings. Cluster 3 had the highest satisfaction, followed by cluster 2, with cluster 1 scoring lowest—demonstrating strong alignment between expert evaluation and user feedback. This correlation suggests that our K-means clustering model effectively distinguishes apps based on their quality and user satisfaction.

To further substantiate our observations, we assessed the statistical significance of the differences between clusters. Wilcoxon tests revealed no significant difference between MARS and user ratings in clusters 1 and 3, indicating alignment between expert and user perceptions. In contrast, cluster 2 showed a statistically significant divergence, suggesting that user satisfaction in this group may depend on different factors than those captured by MARS.

To explore these differences further, we conducted a correlation analysis between the MARS dimensions and weighted average ratings. This helped to identify which MARS dimensions were most closely aligned with user ratings across clusters, revealing distinct priorities for app quality.

In cluster 1, information and engagement emerged as the primary drivers of user satisfaction, with strong positive correlations to user ratings (information=0.74, engagement=0.53). Although functionality had a higher average MARS score (mean 2.89), it showed a negligible negative correlation (−0.08) with user ratings, suggesting that users in this cluster did not perceive added functionality as a key value driver. Aesthetics showed similarly low influence. These patterns may reflect preferences of clinically oriented users, such as health care professionals or informed patients, who prioritize reliable, content-driven tools over design or feature-rich interfaces.

In cluster 2, the significant divergence between metrics, as revealed by the Wilcoxon test, is supported by the correlation analysis. Information quality (mean 3.79, SD 0.30) correlates strongly with user ratings (correlation=0.41), highlighting its importance to users. Although aesthetics received a relatively high MARS score (mean=3.54), it showed the weakest correlation with user ratings (*r*=0.08), suggesting that visual design had little influence on user satisfaction in this cluster. In contrast, engagement (mean=3.36, *r*=0.24) and functionality (mean=3.61, *r*=0.23) both showed modest associations, indicating these dimensions were somewhat more aligned with user expectations. These results suggest that while MARS evaluates certain design and usability features highly, users in this group appear to place greater value on information quality, contributing to the observed divergence between expert and user evaluations.

Cluster 3 shows a borderline difference between MARS scores and user ratings. The correlation analysis reveals that information (mean 4.20, SD 0.36) and aesthetics (mean 4.39, SD 0.37) are the primary factors driving user satisfaction, with strong correlations of 0.62 and 0.58, respectively. Functionality (mean 4.65, SD 0.39) and engagement (mean 4.51, SD 0.36) have moderate to low correlations (functionality=0.39, engagement=0.12). This suggests that while overall alignment exists, users in this cluster place higher value on design and content than interactivity.

Overall, these findings demonstrate that while MARS scores and user ratings often align—particularly in cluster 1—important exceptions exist, most notably in cluster 2, where user feedback may reflect aspects of app quality not fully captured by MARS. By integrating dimension-level MARS scores with user rating correlations, this study offers a more nuanced understanding of the components of app quality that matter most to different user groups. The results highlight that user preferences are not uniform; some users prioritize functionality and efficiency, while others value informational depth or visual appeal. Unlike prior studies by Kim et al [4] and Shen et al [5], which focused primarily on aggregate MARS scores, our use of K-means clustering captures variation in how different quality dimensions resonate with users. This layered approach provides more actionable guidance for tailoring app design to diverse user needs.

### Conclusion

The study assessed 19 mobile apps designed to check for DDIs using the MARS rating scale, with an overall mean MARS score of 3.54. Among the MARS dimensions, information scored the highest, while engagement had the lowest scores and showed the most variability. These results are consistent with the nature of DDI apps, which are primarily used as reference tools where users prioritize quick access to accurate information over prolonged interaction. The positive correlations between all MARS dimensions highlight their interrelatedness, emphasizing the need for well-rounded apps to achieve the most favorable user reviews.

K-means clustering grouped the apps into three distinct clusters: cluster 3, consisting of 5 apps, demonstrated the highest ratings across all dimensions, indicating well-rounded app quality. Cluster 2, with 7 apps, exhibited moderate ratings, reflecting a balanced but less consistent performance. Cluster 1, also with 7 apps, had the lowest ratings, indicating poorer overall performance compared to the other clusters. Scatter plot analysis further revealed that while maintaining strong informational content is important, engagement, functionality, and aesthetics are the key drivers of user perceptions and app quality. These dimensions are critical for distinguishing an app and enhancing user satisfaction.

The comparison of mean weighted app ratings with mean MARS scores confirmed that the K-means clustering model effectively differentiated app quality, with higher MARS scores correlating with greater user satisfaction across clusters. Analyzing the clusters revealed strong alignment between user satisfaction and MARS scores in cluster 1, significant divergence in cluster 2, and slight variation in cluster 3, highlighting differences in how app quality is evaluated. The correlation analysis showed that cluster 1 aligned with MARS scores through engagement and information, while cluster 2 diverged, with users prioritizing information quality. Cluster 3 mostly aligned with MARS scores, favoring well-designed, informative apps, offering valuable insights for developers to better meet user expectations.

In summary, this study offers valuable insights into medication and DDI-checking apps by highlighting the key dimensions that drive user satisfaction. The research introduces an innovative approach by applying K-means clustering to MARS ratings, segregating apps into distinct clusters, and analyzing how different dimensions influence user satisfaction within each cluster. The findings reveal that MARS ratings are cluster-dependent, with dimensions like functionality, engagement, information, and aesthetics playing varying roles depending on the user base. The study also indicates that app ratings received are not uniformly correlated with MARS scores and will differ by cluster based on the specific preferences and needs of different user groups. By uncovering these relationships, the study provides developers with actionable insights to tailor app improvements more effectively according to their targeted user base.

### Limitations

This study has several limitations that should be considered when interpreting the findings. First, it focused exclusively on English-language apps available in the United States, which may limit the global applicability of the findings. Second, mobile apps are dynamic and frequently updated, so our evaluations represent a snapshot in time and may not reflect current app versions. Third, the lack of transparency in app store algorithms affects search reproducibility, as results can vary by device, location, or personalization. Fourth, although rigorous inclusion criteria were applied, the final sample of 19 apps may not capture the full spectrum of DDI-related apps, limiting generalizability. Additionally, the search was limited to the Apple App Store and Google Play Store, which may have excluded relevant apps available through other sources such as web-based tools or alternative platforms, introducing potential sample bias. Fifth, user ratings are dynamic and may be influenced by external factors such as app updates or public sentiment. Sixth, while the MARS tool provides a structured framework, it is based on subjective assessment and may introduce evaluator bias. Two expert raters were used in the evaluation process, which may not fully capture the diversity of professional perspectives. Finally, the study did not include assessments from typical end-users, which may limit the extent to which patient-centered usability perspectives are represented.

### Future Directions

Future studies could expand the analysis by incorporating lay user perspectives through the use of the uMARS tool, which would offer deeper insights into real-world usability and patient-centered experiences. Additionally, integrating specific app features—such as interactive tools, visual aids, and personalization settings—into the clustering model alongside MARS dimensions could enhance the understanding of which functionalities most influence user satisfaction. Given the rapid evolution of mHealth technologies, periodic reassessment of app quality is also recommended to ensure continued relevance and accuracy of evaluations.

## Supplementary material

10.2196/65927 Checklist 1 PRISMA Checklist.

## Abbreviations

DDI: drug-drug interaction
IRR: inter-rater solid reliability
MARS: mobile app rating scale
mHealth: mobile health
PRISMA: Preferred Reporting Items for Systematic Review and Meta-Analysis
SSE: sum of squared errors

## References

[1] Scheife RT, Hines LE, Boyce RD (2015). Consensus recommendations for systematic evaluation of drug-drug interaction evidence for clinical decision support. Drug Saf.

[2] Yee JL, Hasson NK, Schreiber DH (2005). Drug-related emergency department visits in an elderly veteran population. Ann Pharmacother.

[3] Peng Y, Wang H, Fang Q (2020). Effectiveness of mobile applications on medication adherence in adults with chronic diseases: a systematic review and meta-analysis. J Manag Care Spec Pharm.

[4] Kim BY, Sharafoddini A, Tran N, Wen EY, Lee J (2018). Consumer mobile apps for potential drug-drug interaction check: systematic review and content analysis using the mobile app rating scale (MARS). JMIR Mhealth Uhealth.

[5] Shen C, Jiang B, Yang Q (2021). Mobile apps for drug-drug interaction checks in Chinese app stores: systematic review and content analysis. JMIR Mhealth Uhealth.

[6] Stoyanov SR, Hides L, Kavanagh DJ, Zelenko O, Tjondronegoro D, Mani M (2015). Mobile app rating scale: a new tool for assessing the quality of health mobile apps. JMIR mHealth uHealth.

[7] Escriche-Escuder A, De-Torres I, Roldán-Jiménez C (2020). Assessment of the quality of mobile applications (apps) for management of low back pain using the mobile app rating scale (MARS). Int J Environ Res Public Health.

[8] Salazar A, de Sola H, Failde I, Moral-Munoz JA (2018). Measuring the quality of mobile apps for the management of pain: systematic search and evaluation using the mobile app rating scale. JMIR Mhealth Uhealth.

[9] Moher D, Shamseer L, Clarke M (2015). Preferred reporting items for systematic review and meta-analysis protocols (PRISMA-P) 2015 statement. Syst Rev.

[10] Seo S, Cho SI, Yoon W, Lee CM (2022). Classification of smoking cessation apps: quality review and content analysis. JMIR Mhealth Uhealth.

[11] Hallgren KA (2012). Computing inter-rater reliability for observational data: an overview and tutorial. Tutor Quant Methods Psychol.

[12] Siezenga AM, Mertens E, Gelder J van (2023). FutureU - clustering users of a smartphone app-intervention based on usage and engagement. CrimRxiv.

[13] Kodinariya TM, Makwana PR (2013). Review on determining number of cluster in K-means clustering. IJARCSMS.

[14] Anaene Oyeka IC, Ebuh GU, Bernard RJG, Lee MLT (2012). Modified Wilcoxon signed-rank test. OJS.

[15] Agiro A, Bhattacharya A, Khan Z, Tung A, Brahmbhatt Y (2021). PUK13 characteristics of patients receiving sodium zirconium cyclosilicate during hospital-based treatment of hyperkalemia. Value Health.

[16] Rosner B, Glynn RJ, Lee MLT (2006). The Wilcoxon signed rank test for paired comparisons of clustered data. Biometrics.

[17] Schnall R, Rojas M, Bakken S (2016). A user-centered model for designing consumer mobile health (mHealth) applications (apps). J Biomed Inform.

[18] Arthurs N, Tully L, O’Malley G, Browne S (2022). Usability and engagement testing of mhealth apps in paediatric obesity: a narrative review of current literature. Int J Environ Res Public Health.

