# Supplementary material for: Investigating the Quality of Mobile Apps for Drug-Drug Interaction Management Using the Mobile App Rating Scale and K-Means Clustering: Systematic Search of App Stores
Source: JMIR Mhealth Uhealth. 2025 Oct 13;13:e65927. doi: 10.2196/65927 (PMC12516926; doi:10.2196/65927)
Supplement: Checklist 1 [file mhealth-v13-e65927-s001.pdf]

PRISMA 2020 Checklist

Section and Topic	Item #	Checklist item	Location where item is reported
**TITLE**			
Title	1	Identify the report as a systematic review.	Not a formal systematic review; identified as PRISMA-aligned evaluation in Methods.
**ABSTRACT**			
Abstract	2	See the PRISMA 2020 for Abstracts checklist.	Structured abstract provided.
**INTRODUCTION**			
Rationale	3	Describe the rationale for the review in the context of existing knowledge.	Introduction: Para 1–2, rationale linked to app quality uncertainty and variable standards.
Objectives	4	Provide an explicit statement of the objective(s) or question(s) the review addresses.	Introduction: Final paragraph outlines objectives clearly.
**METHODS**			
Eligibility criteria	5	Specify the inclusion and exclusion criteria for the review and how studies were grouped for the syntheses.	Methods: Inclusion/exclusion criteria detailed for app selection.
Information sources	6	Specify all databases, registers, websites, organisations, reference lists and other sources searched or consulted to identify studies. Specify the date when each source was last searched or consulted.	Methods: Sources include Google Play Store and Apple App Store; March 2024.
Search strategy	7	Present the full search strategies for all databases, registers and websites, including any filters and limits used.	Methods: Table 1 provides full list of search terms used.
Selection process	8	Specify the methods used to decide whether a study met the inclusion criteria of the review, including how many reviewers screened each record and each report retrieved, whether they worked independently, and if applicable, details of automation tools used in the process.	Methods: Two reviewers with healthcare background independently screened apps.
Data collection process	9	Specify the methods used to collect data from reports, including how many reviewers collected data from each report, whether they worked independently, any processes for obtaining or confirming data from study investigators, and if applicable, details of automation tools used in the process.	Methods: Data collected independently by two reviewers; MARS evaluations and app metadata.
Data items	10a	List and define all outcomes for which data were sought. Specify whether all results that were compatible with each outcome domain in each study were sought (e.g. for all measures, time points, analyses), and if not, the methods used to decide which results to collect.	Methods: Outcomes include MARS score dimensions and user ratings.
	10b	List and define all other variables for which data were sought (e.g. participant and intervention characteristics, funding sources). Describe any assumptions made about any missing or unclear information.	Methods: Data items include app store metadata; assumptions noted for dynamic rating data.
Study risk of bias assessment	11	Specify the methods used to assess risk of bias in the included studies, including details of the tool(s) used, how many reviewers assessed each study and whether they worked independently, and if applicable, details of automation tools used in the process.	Not applicable; risk of bias assessment not performed for app-based observational data.
Effect measures	12	Specify for each outcome the effect measure(s) (e.g. risk ratio, mean difference) used in the synthesis or presentation of results.	Not applicable; effect measures not used.
Synthesis methods	13a	Describe the processes used to decide which studies were eligible for each synthesis (e.g. tabulating the study intervention characteristics and comparing against the planned groups for each synthesis (item #5)).	Methods: Apps grouped based on inclusion/exclusion; clustering used to analyze MARS synthesis.
	13b	Describe any methods required to prepare the data for presentation or synthesis, such as handling of missing summary statistics, or data conversions.	Not applicable; no imputation or conversion required.
	13c	Describe any methods used to tabulate or visually display results of individual studies and syntheses.	Results: Box plots and scatter plots used for visualization of app dimensions and clusters.

PRISMA 2020 Checklist

Section and Topic	Item #	Checklist item	Location where item is reported
	13d	Describe any methods used to synthesize results and provide a rationale for the choice(s). If meta-analysis was performed, describe the model(s), method(s) to identify the presence and extent of statistical heterogeneity, and software package(s) used.	Methods: K-means clustering, Kruskal-Wallis, and Wilcoxon tests detailed for synthesis.
	13e	Describe any methods used to explore possible causes of heterogeneity among study results (e.g. subgroup analysis, meta-regression).	Methods: Cluster-level correlations explored to identify heterogeneity in user satisfaction.
	13f	Describe any sensitivity analyses conducted to assess robustness of the synthesized results.	Not applicable; no sensitivity analyses performed.
Reporting bias assessment	14	Describe any methods used to assess risk of bias due to missing results in a synthesis (arising from reporting biases).	Not applicable.
Certainty assessment	15	Describe any methods used to assess certainty (or confidence) in the body of evidence for an outcome.	Not applicable.
**RESULTS**			
Study selection	16a	Describe the results of the search and selection process, from the number of records identified in the search to the number of studies included in the review, ideally using a flow diagram.	Results: PRISMA-style flow diagram provided (Figure 1).
	16b	Cite studies that might appear to meet the inclusion criteria, but which were excluded, and explain why they were excluded.	Results: Apps excluded based on disease-specific focus or lack of interaction checking.
Study characteristics	17	Cite each included study and present its characteristics.	Results: App characteristics and scores summarized in Results and supplementary table.
Risk of bias in studies	18	Present assessments of risk of bias for each included study.	Not applicable; no formal bias assessment tool used.
Results of individual studies	19	For all outcomes, present, for each study: (a) summary statistics for each group (where appropriate) and (b) an effect estimate and its precision (e.g. confidence/credible interval), ideally using structured tables or plots.	Results: Scores and correlations for each app dimension presented.
Results of syntheses	20a	For each synthesis, briefly summarise the characteristics and risk of bias among contributing studies.	Results: Apps grouped into clusters; bias not assessed.
	20b	Present results of all statistical syntheses conducted. If meta-analysis was done, present for each the summary estimate and its precision (e.g. confidence/credible interval) and measures of statistical heterogeneity. If comparing groups, describe the direction of the effect.	Results: Means and correlation coefficients reported; clustering validated statistically.
	20c	Present results of all investigations of possible causes of heterogeneity among study results.	Results: Cluster analysis revealed inter-cluster variation in user preferences.
	20d	Present results of all sensitivity analyses conducted to assess the robustness of the synthesized results.	Not applicable.
Reporting biases	21	Present assessments of risk of bias due to missing results (arising from reporting biases) for each synthesis assessed.	Not applicable.
Certainty of evidence	22	Present assessments of certainty (or confidence) in the body of evidence for each outcome assessed.	Not applicable.
**DISCUSSION**			
Discussion	23a	Provide a general interpretation of the results in the context of other evidence.	Discussion: Interprets findings in context of Kim et al. and Shen et al.
	23b	Discuss any limitations of the evidence included in the review.	Discussion: Limitations include sample size, dynamic ratings, lack of reproducibility.
	23c	Discuss any limitations of the review processes used.	Discussion: Notes limitations of app store search algorithms (e.g., lack of transparency, variability in

PRISMA 2020 Checklist

Section and Topic	Item #	Checklist item	Location where item is reported
			results), regional/language restrictions, and the limited number of expert reviewers used for MARS assessments.
	23d	Discuss implications of the results for practice, policy, and future research.	Discussion: Recommends targeted development based on user-cluster preferences.
**OTHER INFORMATION**			
Registration and protocol	24a	Provide registration information for the review, including register name and registration number, or state that the review was not registered.	Review was not registered.
	24b	Indicate where the review protocol can be accessed, or state that a protocol was not prepared.	No formal protocol was prepared.
	24c	Describe and explain any amendments to information provided at registration or in the protocol.	Not applicable.
Support	25	Describe sources of financial or non-financial support for the review, and the role of the funders or sponsors in the review.	Funding Statement: No external funding received.
Competing interests	26	Declare any competing interests of review authors.	Conflict of Interest: None declared.
Availability of data, code and other materials	27	Report which of the following are publicly available and where they can be found: template data collection forms; data extracted from included studies; data used for all analyses; analytic code; any other materials used in the review.	Data Availability section included; analysis conducted in RStudio; no public dataset shared.

From: Page MJ, McKenzie JE, Bossuyt PM, Boutron I, Hoffmann TC, Mulrow CD, et al. The PRISMA 2020 statement: an updated guideline for reporting systematic reviews. BMJ 2021;372:n71. doi: 10.1136/bmj.n71. This work is licensed under CC BY 4.0. To view a copy of this license, visit https://creativecommons.org/licenses/by/4.0/
